# Transcriptomic Profile Reveals Deregulation of Hearing-Loss Related Genes in Vestibular Schwannoma Cells Following Electromagnetic Field Exposure

**DOI:** 10.3390/cells10071840

**Published:** 2021-07-20

**Authors:** Alessandra Colciago, Matteo Audano, Veronica Bonalume, Valentina Melfi, Tasnim Mohamed, Adam J. Reid, Alessandro Faroni, Peter A. Greer, Nico Mitro, Valerio Magnaghi

**Affiliations:** 1 Department of Pharmacological and Biomolecular Sciences, Università Degli Studi di Milano, Via G. Balzaretti 9, 20133 Milan, Italy; alessandra.colciago@unimi.it (A.C.); Matteo.Audano@unimi.it (M.A.); veronica.bonalume@unimi.it (V.B.); valentina.melfi@unimi.it (V.M.); tasnim.mohamed@unimi.it (T.M.); nico.mitro@unimi.it (N.M.); 2 Blond McIndoe Laboratories, Division of Cell Matrix Biology and Regenerative Medicine, School of Biological Sciences, Faculty of Biology Medicine and Health, University of Manchester, Manchester Academic Health Science Centre, Manchester M13 9NQ, UK; Adam.Reid@manchester.ac.uk (A.J.R.); alessandro.faroni@manchester.ac.uk (A.F.); 3 Department of Plastic Surgery & Burns, Wythenshawe Hospital, Manchester University NHS Foundation Trust, Manchester Academic Health Science Center, Manchester M13 9NQ, UK; 4 Department of Pathology and Molecular Medicine, Queen’s University, Kingston, ON K7L 3N6, Canada; greerp@queensu.ca

**Keywords:** Schwann cell, NF2, GJB2, hearing loss, NEFL, TPRN, OTOGL, REST

## Abstract

Hearing loss (HL) is the most common sensory disorder in the world population. One common cause of HL is the presence of vestibular schwannoma (VS), a benign tumor of the VIII cranial nerve, arising from Schwann cell (SC) transformation. In the last decade, the increasing incidence of VS has been correlated to electromagnetic field (EMF) exposure, which might be considered a pathogenic cause of VS development and HL. Here, we explore the molecular mechanisms underlying the biologic changes of human SCs and/or their oncogenic transformation following EMF exposure. Through NGS technology and RNA-Seq transcriptomic analysis, we investigated the genomic profile and the differential display of HL-related genes after chronic EMF. We found that chronic EMF exposure modified the cell proliferation, in parallel with intracellular signaling and metabolic pathways changes, mostly related to translation and mitochondrial activities. Importantly, the expression of HL-related genes such as NEFL, TPRN, OTOGL, GJB2, and REST appeared to be deregulated in chronic EMF exposure. In conclusion, we suggest that, at a preclinical stage, EMF exposure might promote the transformation of VS cells and contribute to HL.

## 1. Introduction

Vestibular schwannoma (VS), also known as acoustic neuroma, is a benign tumor of the VIII cranial nerve, which arises from myelin-forming Schwann cells (SCs), most commonly in the superior branch of the vestibular nerve [1]. About 90% of VSs are sporadic and unilateral, for which pathogenic causes are still poorly understood [2]. However, about 5% of VS is bilateral and occurs in neurofibromatosis type 2 (NF2), an autosomal dominant genetic disease [3]. The NF2 tumor suppressor gene, coding for the protein merlin, is inactivated in both sporadic and NF2-associated VS [4]. Merlin is a tumor suppressor, able to integrate different mechanisms and deputed to regulate signaling pathways contributing to cell proliferation, adhesion, motility, and survival [5,6]; therefore, mutations in NF2 are strongly associated with SC oncotransformation [7,8].

VSs are slow-growing brain tumors that negatively impact quality of life. More than 90% of NF2 patients demonstrate hearing impairments on the side of a VS [2], with audiogram assessment revealing a high-frequency slope in more than 60%. The mechanisms underlying hearing loss (HL) in NF2 are still unclear and are presumed to be multifactorial. HL is the most common sensory disorder in the world population, affecting around 40 million people in the U.S. Among causes of sensorineural HL, the structural and physiological dysfunction of VIII cranial nerve has a high prevalence [9]. Hence, it is conceivable that changes in the biological and/or pro-oncogenic properties of SCs may affect VS onset and related HL.

Several studies have reported an increasing incidence of VSs over the last decades [10], likely due to improved diagnostic capability. However, increased exposure to possible risk factors including non-ionizing radiation and electromagnetic field (EMF) have also been considered as pathogenic causes. Thus, rapid and wide increases in the use of mobile and cordless phones raised concerns about the increased risk for VS and subsequent HL [11,12]. Data collected from case-control and case-case studies corroborated this pathogenic association [12,13].

In this light, our findings indicated that EMF-exposed SCs changed their biological features (e.g., morphology, proliferation, migration, and myelinating capability), modifying their phenotype toward a proliferative/migrating state [7,14]. In these cells, the oncosuppressor merlin is downregulated, leading to activation of the intracellular MAPK/ERK-PI3/Akt and Hippo signaling pathways. We propose that these SC changes might be pathologically relevant for the development of VS, as a cause of HL. We suggest that the EMF exposure represents a second hit, affecting SC development in predisposed and susceptible human subjects (specifically, those bearing NF2 mutations or changes in merlin expression) prone to developing VS and subsequent HL.

Here, we analyzed the molecular mechanisms underlying human SC biologic changes and/or oncogenic transformation following EMF exposure, which might be potentially responsible for VS development and HL. Using NGS and differential display transcriptomic analyses, we characterized the gene profiles of VS cells and asked whether novel or HL-related genes may be differentially affected by EMF. We found that chronic exposure to EMF altered some important intracellular and metabolic pathways, suggesting an impact on the transformation of VS cells and progression to HL.

## 2. Materials and Methods

### 2.1. Cell Cultures

HEI-193 is a human VS cell line derived from a NF2 patient [15,16]. These cells have a mutation in the *NF2* gene that results in defective splicing of the NF2 mRNA, leading to the production of a C-terminally truncated merlin protein, so that no or very low levels of merlin protein is produced. The cells were plated in Dulbecco’s modified Eagle’s medium (DMEM, Euroclone, Pero, Italy) with 10% fetal bovine serum (FBS; Gibco-Life Technologies, Milan, Italy), ±forskolin (fsk; Sigma-Aldrich, Milan, Italy), at different concentrations and times.

Human nerves were isolated from a patient participant undergoing reconstructive surgery at Wythenshawe Hospital, Manchester University NHS Foundation Trust, the UK, after informed consent was obtained from all subjects involved in the study. All procedures were approved by the National Research Ethics Committee, the UK (NRES 18/NW/0847) and conformed with the World Medical Association Declaration of Helsinki. Primary naïve human SC were obtained as per Piovesana et al. [17]. Briefly, nerves were dissected, and single extracted fibers were cut into small pieces, then cultured in 60 cm^2^ dishes with SC media supplemented with 10 µM fsk and 100 ng/mL glial growth factor 2 (GGF-2, Acorda Therapeutics, Ardsley, NY, USA) for two weeks. Nerves were digested with dispase (Life Technologies, Carlsbad, CA, USA) and collagenase IV (Life Technologies) for 24 h, then the solution was gently triturated and passed through a sterile 70 µm mesh. After centrifugation, the cell pellet was gently resuspended in SC media supplemented with 10 µM fsk + 100 ng/mL GGF-2 and seeded onto poly-d-lysine-coated (Sigma-Aldrich) plates for the experiments. Overall, the appearance and growth characteristics of all cells used in this study were compared with published information to ensure their authenticity.

### 2.2. EMF Treatment

Cells were exposed to 50 Hz 0.1 T EMF (at 37 °C) for different protocols: (1) one 10-min single treatment, mimicking the acute exposure; and (2) 10-min treatment per day for 5 days (every 24 h at the same time) mimicking the chronic exposure. The EMF was produced by the magnetic field generator (Ugo Basile, Gemonio, Italy). The cells were then analyzed at different time points according to the specific assays. Cells used as controls were plated in the same culture conditions without EMF exposure.

### 2.3. Immunofluorescence (IFL) and Cell Characterization

Microscopy and IFL were used for SC morphologic characterization. An antibody for the specific SC marker S100 (Dako Agilent, Santa Clara, CA, USA) was used. S100 stains cells of neural origin and is characteristic of SCs in their early stages of development/differentiation. SC cytoskeletons were stained with phalloidin (Sigma-Aldrich). Cells were plated on coverslips, then fixed 20 min in 4% paraformaldehyde (Sigma-Aldrich) and washed in phosphate buffer saline (PBS, Euroclone, Pero, Italy). Cells were permeabilized with 0.2% Triton X-100 (Sigma-Aldrich) and blocked for 1 h with 0.25% BSA (Sigma-Aldrich), depending on the host species of the secondary antibody. Primary antibodies to S100 (1:150) and phalloidin (1:300) were applied overnight at 4 °C in a humidified chamber. The following day, slides were rinsed in PBS and incubated in the FITC Alexa-488-conjugated secondary antibody (Thermo Fisher Scientific, Monza, Italy), washed, and mounted using Vectashield^TM^ plus DAPI for nuclear staining (Vector Laboratories, Oxfordshire, UK). Negative controls lacking primary antibodies were also performed. Confocal laser scanner microscopy was performed by the Zeiss Confocal System and Zen software analysis (Zeiss, Oberkochen, Germany).

### 2.4. In Vitro Cell Proliferation, Viability, and Migration Assays

Cells were plated in Petri dishes and analyzed for viability, proliferation, and migration. All measurements were done by using ImageJ 1.51 (NIH, Bethesda, MD, USA) software. Approximately 6 × 10^4^ cells were plated into 35 mm Petri dishes and analyzed after 6, 24, 48, 72, 96, and 120 h. To assess proliferation, the cells collected with Trypsin 0.05%-EDTA 0.02% in PBS were then resuspended in DMEM and counted with a hemocytometer. Viability was tested by the MTT (3-(4,5-dimethylthiazol-2-yl)-2,5-diphenyltetrazolium bromide; Sigma-Aldrich) assay. Cells were seeded in 35 mm Petri dishes, then stained with MTT solution (0.5 mg/mL) for 30 min at 37 °C. Absorbance was measured at 570 nm. Each experimental point was in quadruplicate and experiments were replicated at least three times; data were expressed as absorbance ± SEM of the mean. The migration assay was performed by the wound healing assay, making a wound scratch on the cell monolayer. Cells were cultured with very low serum concentration to avoid the effect of cell proliferation. Cells were photographed with a light microscope (Axiovert 200 Zeiss) at different time points (6, 24, 48, and 72 h) after the scratch at the final magnification of 600×. Images were acquired using MetaVue software and the 2D area covered by the cell migration was measured. Each experimental point was in triplicate. Each data point was calculated as the difference of the 2D scratched area (at time 0) minus the 2D remaining area at each time point. Data were expressed in μm^2^ ± SEM of the mean.

### 2.5. RNA Preparation and qRT-PCR Analysis

RNA was extracted using Trizol (Gibco-Life Technologies) according to the manufacturer’s protocol, then quantified with Nano-Drop2000 (Thermo Scientific, Waltham, MA, USA). Pure RNA was obtained after DNase I treatment (Sigma-Aldrich). The RT-reaction was carried on with RT iScript Supermix 5× (Bio-Rad, Segrate, Italy) on 1 μg of purified RNA. The product was used to perform qRT-PCR assays using gene specific primers: P0, 5′-CCTGCTCTTCTCTTCTTTG-3′ and 5′-CACAGCACCATAGACTTC-3′; PMP22: 5′-TCCTGTTCCTTCACATCG-3′ and 5′-TGCCAGAGATCAGTCCTG-3′; NF2: 5′-ACGATGGCCAATGAAGCTCTGATG-3′ and 5′-TGGCCTTGATTCGCTGCATCTC-3′; glyceraldehyde-3-phosphate dehydrogenase (GAPDH) was used as the housekeeping gene. qRT-PCR was performed by measuring the incorporation of SYBR Green dye (Bio-Rad) on a CFX 96 Real Time System-C1000 touch thermal cycler (Bio-Rad). Data analysis was performed by the CFX Manager 2.0 software (Bio-Rad) using the 2^−ΔΔCt^ method for relative quantification. The Pfaffl method was used to compare the experimental samples, normalized to the mean levels of the housekeeping gene [18].

### 2.6. Statistical Analysis

Data were statistically evaluated by GraphPad Prism 8.00 (San Diego, CA, USA), using the parametric *t*-test and two-way ANOVA with Sidak’s post-test.

### 2.7. NGS Technology RNA-Seq

RNA-Seq was performed on HEI-193 cells chronically exposed to EMF and control cells (CTRL) that were not exposed to EMF. For each condition, experiments were done in triplicate in order to perform statistics for differential expression studies. For each sample, quantity control was performed using an Agilent Bioanalyzer (Santa Clara, CA, USA) to validate RNA integrity. Each sample of 500 ng total RNA was processed to obtain a library of indexed fragments using an Illumina Stranded mRNA Kit (San Diego, CA, USA). Equimolar quantities of indexed libraries for each sample were loaded for sequencing on two flow cells, Illumina MidOutput 150 cycles, and sequenced using Illumina NextSeq550dx (RUO mode). About 40–60 × 10^6^ fragments of each sample were sequenced in paired-end (PE) modality, which characterized 75 nucleotides of both ends of each fragment of the library. The raw data were generated as fasta.gz. For quality control of sequencing, two rounds of reading generated optimal metrics, with >85% of reads passing the filtering and 93% of reads with Q score > 30; technical error rate was 0.32%. Thus, no problems in sequencing occurred. Another quality control of reads was done using the tool FastQC (v 0.11.8, Babraham Bioinformatics, Babraham Institute, Cambridge, CB22 3AT, UK), which analyzed some of the other parameters including the multiplex balance of samples, distribution of specific characteristics of each base, representativeness of nucleotides, mean size of reads, and presence of adapters.

### 2.8. Bioinformatic Analysis of Transcriptome

The sequence reads were aligned with the human genome (Build GRCh38) to be converted into quantifications of different transcripts present in the samples. The software Salmon (v.0.13.1) [19] was utilized. Pairing percentage was around 90%. The outputs from Salmon represented a quantification of the transcript expression present in the reference genome, which must be then normalized for analysis. The transcriptional analysis was performed with DeSeq2 software using the library R DESeq2 (v.1.24.0) [20]. At the beginning, a selection of all transcripts was performed to exclude those not expressed in our conditions and selecting only the transcripts where the sum of six expression values produced more than 20 reads. Consequently, the analyzed dataset was reduced to 20,190 transcripts. The box plot analysis revealed the expression values (in log2 scale) for each sample, which did not produce false data. We also performed GSEA analysis, which is a commonly used statistical method that analyzes a lengthy list of deregulated genes and takes into consideration small, though coordinated, changes in expression.

RNA-Seq files (Fastq data and quantitation matrix) were deposited in the GEO (gene expression omnibus) database. The access number is GSE174389, and the security token is ixovwimkrlebdkf.

## 3. Results

### 3.1. Characterization of Human Schwannoma-Derived Cells

**Figure 1:**
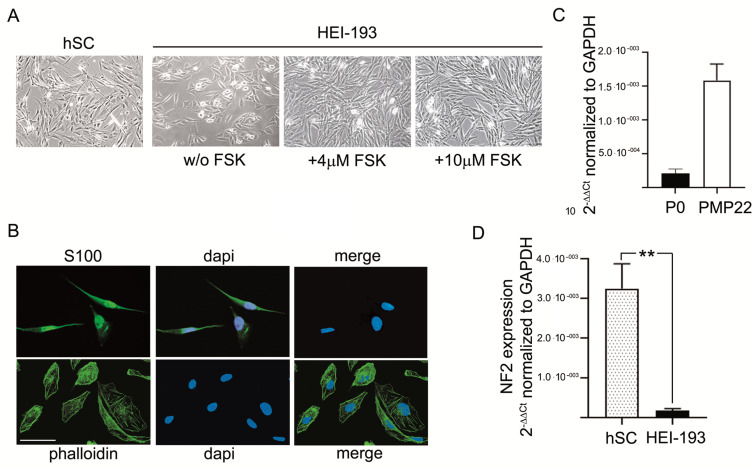
Characterization of HEI-193 cells. (**A**) Representative phase-contrast images of primary human SCs (hSC) and HEI-193 cells in culture at 7 div (day in vitro), following treatment with 4 and 10 μM forskolin (fsk). Scale bar 10 μm. (**B**) IFL microscopy images of HEI-193 characterized by immunopositivity for the S100 marker (anti-s100-488, in green), showing a cell purity more than 98%. Cells were typically spindle-shaped. The actin cytoskeleton was assessed by labelling for f-actin (phalloidin-FITC, in green). Nuclei were stained with Dapi, in blue. Scale bar 10 µm. (**C**) Relative quantification by qRT-PCR of mRNAs levels, coding for proteins P0 and PMP22, respectively, in HEI-193 cells. Data were normalized to the housekeeping gene GAPDH and expressed as 2^−ΔΔCt^. The columns were expressed as fold changes. The values are means ± S.D. (*n* = 4). (**D**) Merlin (NF2) mRNA levels were assayed by qRT-PCR, showing a significant decrease (** *p* < 0.01) in HEI-193 cells versus hSCs. Data were normalized to the housekeeping gene GAPDH and expressed as 2^−ΔΔCt^. The columns were expressed as fold changes. The values are means ± S.D. (*n* = 4).

The HEI-193 human VS cell line [15], derived from an NF2 patient, was used for the experiments. These cells showed a SC-like morphology, with the characteristic spindle-shape in vitro, resembling naïve primary hSCs (Figure 1A). Treatment of HEI-193 cells with fsk (4 or 10 μM) induced a pronounced morphologic differentiation toward the naïve SC phenotype after five days in culture (Figure 1A). The HEI-193 cells were characterized for immunopositivity against S100 (typical SC marker) and phalloidin labelling (Figure 1B), corroborating a previous publication [21] and confirming the SC-like morphology. Basal gene expression of typical myelin proteins P0 and PMP22 (characteristic of SCs) indicated that HEI-193 maintains the SC-like phenotype (Figure 1C). Interestingly, the HEI-193 VS genotype, being defective NF2 cells, should not express consistent levels of the tumor suppressor merlin. As expected, indeed, these cells expressed very low levels of the NF2 transcript (nearby zero), about 10^−2^ orders of magnitude (*p* < 0.01) less than naïve hSCs (Figure 1D).

### 3.2. Chronic Exposure to EMF Induces Proliferative Changes in VS Cells

**Figure 2:**
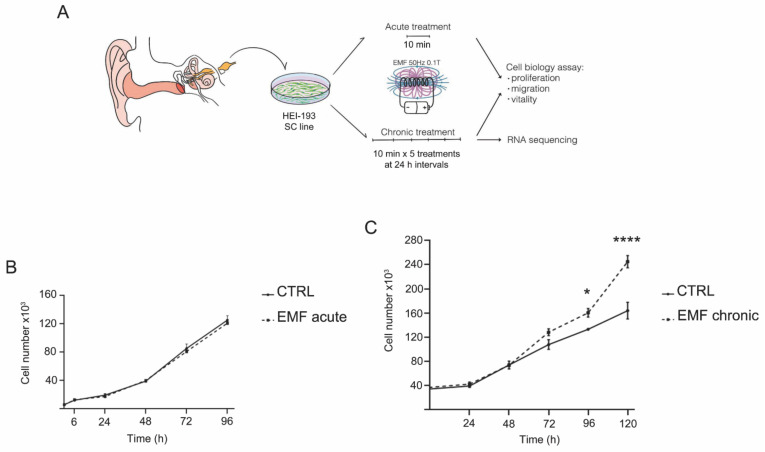
Proliferation effects on HEI-193 cells exposed to acute and chronic EMF. (**A**) Scheme of the experimental protocol applied. HEI-193 cells were exposed to EMF of 50 Hz, 0.1 T, for one 10-min single treatment (acute protocol) or for 10-min treatment/per day for five days (every 24 h at the same time; chronic protocol). Then, the cells were assayed for proliferation, migration, vitality, and NGS sequencing. (**B**) Proliferation was assessed at 6, 24, 48, 72, and 96 h, following a single acute EMF exposure. Experiments were repeated at least three times and data expressed as cell number ± SEM of the mean. (**C**) Proliferation was assessed at 24, 48, 72, 96, and 120 h, following a five day chronic EMF exposure. EMFs produced a significant increase in cell proliferation at 96 (* *p* < 0.05) and 120 (**** *p* < 0.0001) h. Experiments were repeated at least three times and data expressed as cell number ± SEM of the mean. Two-way ANOVA using Sidak’s post-hoc test was used for statistical analysis.

To test the effects of EMF on HEI-193 biological features, we applied either an acute EMF treatment of 50 Hz, 0.1 T for 10 min or a chronic treatment consisting of the same treatment repeated for five consecutive days (Figure 2A). When cell proliferation was assessed, the acute EMF exposure did not induce any significant difference in HEI-193 cell proliferation compared to the untreated control cells (Figure 2B). However, when the cells were subjected to chronic EMF, we observed increased HEI-193 cell proliferation at 72 and 96 h (*p* < 0.05), then found a higher significant rise in proliferation at 120 h (*p* < 0.0001) following the first exposure (Figure 2C). These results indicate that multiple EMF exposures are associated with increased proliferation rates in these NF2-deficient cells.

### 3.3. Chronic EMF Exposure Effect on Viability and Migration of VS Cells

**Figure 3:**
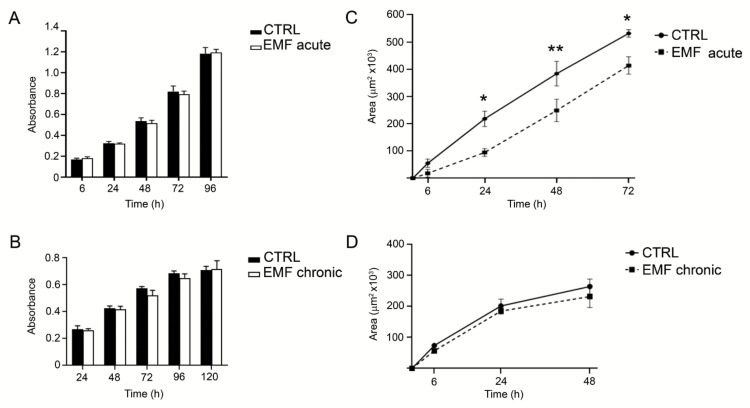
Migration and viability effects on HEI-193 cells exposed to acute and chronic EMF. (**A**) Cell viability was assessed by the MTT assay at 6, 24, 48, 72 and 96 h following a single acute EMF exposure. Each experimental point was in quadruplicate and experiments replicated at least three times; data were expressed as absorbance ± SEM of the mean. (**B**) Cell viability was assessed at 24, 48, 72, 96, and 120 h, following completion of a 5-day chronic EMF exposure. Each experimental point was in quadruplicate and experiments replicated at least three times; data were expressed as absorbance ± SEM of the mean. (**C**) Cell migration was assessed at 6, 24, 48, and 72 h, following a single acute EMF exposure. EMF treatment was associated with a significant decrease in cell migration at 24 (* *p* < 0.05), 48 (** *p* < 0.01), and 72 (* *p* < 0.05) h. Experiments were repeated at least three times. Each data point was calculated as the difference of the 2D scratched area (at time 0) minus the 2D remaining area at each specific time point, representing the 2D area covered by the cell migration. Data were expressed in μm^2^ ± SEM of the mean. (**D**) Cell migration was assessed at 6, 24, and 48 following a chronic EMF exposure. Experiments were repeated at least three times. Each data point was calculated as the difference of the 2D scratched area (at time 0) minus the 2D remaining area at each specific time point, representing the 2D area covered by the cell migration. Data were expressed in μm^2^ ± SEM of the mean. Two-way ANOVA using Sidaki’s post-hoc test was used for statistical analysis.

We next tested the effects of acute or chronic EMF exposure on HEI-193 cell viability and migration. HEI-193 cells remained viable and increased in number after either acute (Figure 3A) or chronic (Figure 3B) exposure to the same degree as the control unchallenged cells.

Interestingly, migration of HEI-193 cells was significantly reduced (starting from 24 and lasting to 72 h) after acute EMF (Figure 3C), but not after chronic exposure (Figure 3D).

### 3.4. Identification of Differentially Expression Genes (DEG) in HEI-193 Cells Following Chronic EMF Exposure

**Figure 4:**
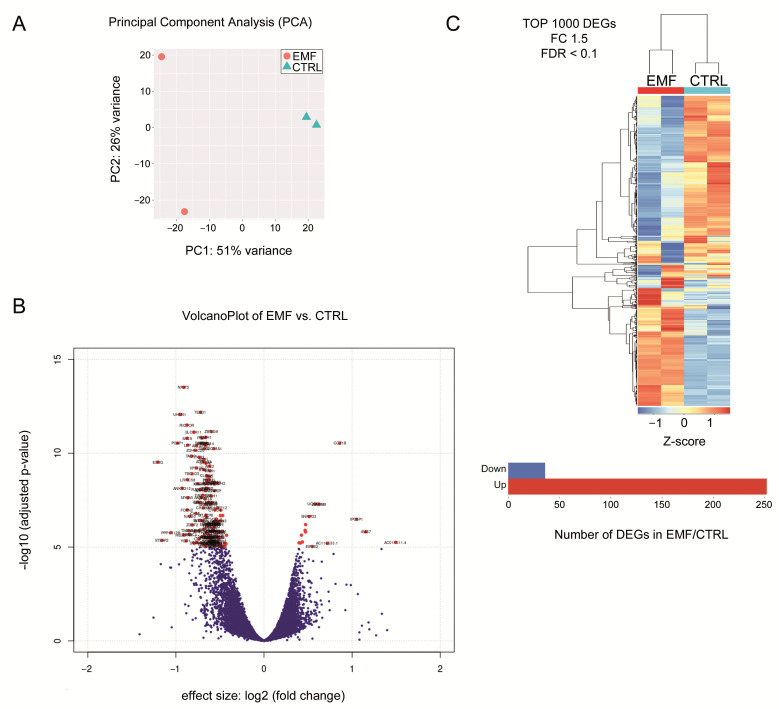
Identification of DEG in HEI-193 cells following chronic EMF. (**A**) Principal component analysis (PCA) indicated that PC#1 included 51% of system variability and this tends to separate the sample replicates of EMF condition. Indeed, PC#2 included 26% of system variability. (**B**) Volcano plot displaying DEGs between the control (CTRL) and EMF-exposed cells. The vertical axis (y-axis) corresponds to the mean expression value of log 10 (*q*-value), and the horizontal axis (x-axis) displays the log 2 (fold change) value. The red dots represent the upregulated expressed transcripts; the blue dots represent the transcripts whose expression is downregulated. Positive *x*-values represent upregulation and negative *x*-values represent downregulation. (**C**) Heat map of the 1000 top upregulated genes in VS cells from CTRL versus EMF exposed. DEGs were selected setting a 1.5 fold change with a *p*-value of 0.01.

The HEI-193 cells were next analyzed for transcriptomic changes after chronic EMF exposure by NGS RNA-Seq. Principal component analysis (PCA), a computational technique based on complexity reduction and maximization of differences among samples, tested the quality of the data and the distribution of samples based on their transcriptomic profiles (Figure 4A). Higher plotted distances between the different experimental conditions (EMF versus control) would be the difference in terms of transcriptional profile; again, the more the replicates are similar (reproducibility), the dots would plot even more in the same PCA region. It is evident that PC#1 included 51% of system variability (Figure 4A), and this tends to separate the sample replicates of EMF condition (left side) from replicates of the control condition (CTRL, right side).

Sample replicates of each condition (CTRL vs. EMF) were then used to identify DEG. This analysis was performed on 20,190 transcripts above the defined detection limit (sum > 20), then normalized and compared between the control and EMF treated groups. A fold-change based *t*-test was used for statistical analysis. To increase the number of differentially expressed transcripts, two lists, one with fold change 2 and the other setting with fold change 1.5, were considered (Supplementary Figures S1 and S2, respectively). Changing the detection limit (sum > 15), normalized, and compared per groups control vs. EMF, we found 19,881 transcripts changed. The differentially expressed transcripts were then selected setting a 1.5 fold change with a *p*-value of 0.01. A VolcanoPlot analysis was performed, comparing EMF vs. the control samples (Figure 4B). Many transcripts appeared significantly downregulated (red dots, left side) following chronic EMF, whereas other transcripts were significantly upregulated (red dots, right side). On a hierarchical clustering plot, the identified top 1000 DEG distinguished the control from the EMF-exposed samples (Figure 4C). In particular, setting the 1.5 fold change with a *p*-value of 0.01, we found 55 DEGs. Interestingly, dynein heavy chain 17, proprotein convertase subtilisin/kexin type 1 (PCSK1), and thyroglobulin were upregulated in the EMF-exposed samples, while transforming growth factor alpha (TGFalpha), and Prader Willi/Angelman region RNA 5 (PWAR5) were downregulated in the EMF cells.

### 3.5. Chronic EMF Exposure Changed Metabolic Pathways of HEI-193 Cells

The bioinformatic analysis of RNA-Seq raw data was performed with the gene set enrichment analysis (GSEA) plot, giving score curves. GSEA was performed with the canonical pathways and biological process gene sets in the GSEA Molecular Signatures Database. “Signal-to-noise” ratio (SNR) statistic was used to rank the genes as per their correlation with either the EMF exposure or the control groups. GSEA analysis produced a full list of the rank-ordered group of genes participating in the top positively or negatively enriched pathways. The whole analysis indicated that following chronic EMF exposure, about 40 complex and important intracellular signaling and metabolic pathways were changed in HEI-193 cells. In detail, the pathway analysis highlights the major biological processes altered with the chronic EMF exposure (CTRL VS EMF). The higher the normalized enrichment score (NES), the higher the ranking and the statistical significance of the pathway. The class of genes clustered for each pathway are reported in Table 1. Translational and ribosomal pathways were the most significantly changed in HEI-193 cells chronically exposed to EMF; for instance, protein targeting to ER or translational termination as well as ribosomal assembly were upregulated (blue arrows in Table 1). In addition, mitochondrial translational elongation and termination were upregulated by EMF (red arrows in Table 1). Interestingly, all these pathways are complex systems controlling the fundamental biologic mechanisms for cell metabolism. A further detailed investigation is on-going to confirm these changes.

### 3.6. Hippo Pathway- and HL-Related Gene Expression Changes in HEI-193 Cells Following Chronic EMF Exposure

**Figure 5:**
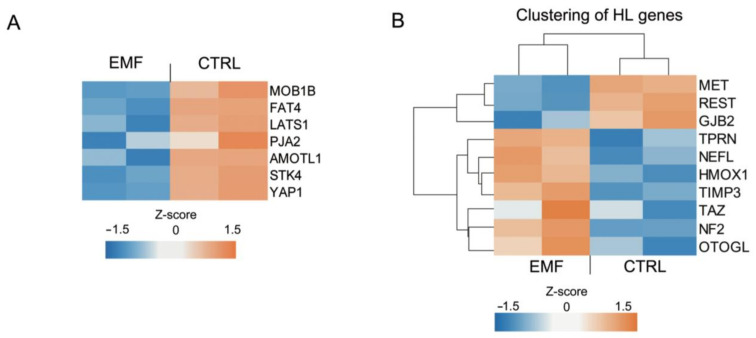
Identification of Hippo and HL-related DEGs in HEI-193 cells following chronic EMF. (**A**) Clustering of the Hippo-related genes that are deregulated in VS cells from control (CTRL) versus EMF exposed. (**B**) Clustering of the HL-related genes that are deregulated in VS cells from CTRL versus EMF exposed.

Previously, we demonstrated that Hippo signaling, which is important for SC oncotransformation, and some of the regulatory proteins belonging to this pathway are targeted by EMF [7]. Indeed, at least 21 genes encoding upstream or downstream mediators of Hippo signaling were altered, mostly downregulated. Herein, we confirmed that some proteins of the Hippo signaling pathway were altered in HEI-193 cells following chronic EMF exposure (Figure 5A). The heatmap visualizes the genes contributing to the Hippo pathway enrichment, which are mostly downregulated by the chronic EMF (Figure 5A). Genes coding for proteins involved in cell polarity such as angiomotin like protein (Amotl), or involved in cell adhesion and myelinogenesis such as the cadherin protein (Fat), displayed decreased expression following chronic EMF exposure (blue square) versus the controls (CTRL, brownish squares). In addition, Yes-associated protein 1 (Yap1), which mediates apoptotic and proliferative effects in SCs, was found to be downregulated in HEI-193 cells, following chronic EMF exposure (Figure 5A). Overall, this analysis confirmed that some regulatory proteins of the Hippo pathway regulate VS cell fate following chronic EMF exposure.

Finally, we found that several DEGs detected in our EMF-induced differential expression profile matched with a set of genes known to be involved in the most important HL and sensorineural HL diseases [22,23] (Figure 5B). NEFL (a neuron-specific intermediate filament essential for the radial growth of axons), TPRN (taperin), HMOX1 (heme oxygenase1), and OTOGL (otogelin-like protein) were upregulated, while GJB2 (connexin32, Cx32) and REST (a DNA-binding protein that complexes the histone deacetylases) were downregulated following chronic EMF exposure.

## 4. Discussion

Our findings show that chronic EMF exposure has a strong impact on VS cells, affecting their biomolecular characteristics. The EMF exposure induced a differential expression of several genes and biochemical pathways, mostly related to ribosomal and translational activation. Importantly, several HL-related genes were among those altered by the EMF exposure, although the physiological significance of these changes require further investigation.

The reliability of our NF2 defective cellular model was supported by the HEI-193 phenotype, which is in accordance with the cell characteristics already published by Hung et al. [15,16]. Indeed, these cells are a good paradigm of SCs phenotype, bearing the characteristic spindle-shaped morphology and expression of known SC markers [21,24].

Among all genes found to be dysregulated in VS cells following EMF chronic exposure, dynein, proprotein convertase subtilisin/kexin type 1 (PCSK1), and thyroglobulin were upregulated, while TGFα and the Prader Willi/Angelman region 5 (PWAR5) lncRNA gene were downregulated. Proteins involved in microtubule dynamics and axonal transport such as dynein are essential for the bidirectional transport of cargos including organelles and mRNA between soma and synaptic terminals. In this light, dynein is important in nerve regeneration [25,26] as well as in regulating injury-induced SC remodeling and myelination [27]. However, a direct role for dynein in SC transformation has not been elucidated. One hypothesis for dynein involvement in the mechanobiology of VS cells and remodeling is strengthened by the observation that dynein drives nuclear localization of Yap, and regulation of myofibroblast differentiation [28]. PCSK1 belongs to the pro-protein convertase family of proteases that are involved in the processing of precursor proteins into their diverse active end-products. PCSK1 is strongly induced in injured nerves and in SCs [29]. Although TGFα does not have a clear function in PNS cells, its role in VS and immune mediated HL has recently been described [30]. TGFα has been identified as an ototoxic molecule [31], thereby its decrease might also be protective.

Our findings also corroborate some findings of another transcriptomic analysis performed on VS [32], which identified mTOR and PI3K as the principal signaling pathways implicated in schwannoma onset [33,34,35]. Changes in the expression of components of the Hippo signaling pathway also corroborate our previous observations [7], showing the putative involvement of this pathway in SC oncotransformation to VS, likely contributing to HL. Previously, we showed that acute exposure of rat SCs to EMF contributes to oncotransformation. EMFs induced changes in SC NF2/merlin expression, cell migration, chemotactic responsivity, and cytoskeleton reorganization [7]. We showed MAPK/Erk activation involved in SC proliferation as well as activation of Hippo/YAP signaling, which are commonly altered during tumorigenesis. We also found that some genes, known to be upstream or downstream mediators of Hippo (Amotl2, Dchs, Fat, Wnt1), were changed. Genes coding for proteins involved in cell polarity such as Amotl, or involved in cell adhesion and myelinogenesis such as cadherin protein Fat, or mediating apoptotic and proliferative effect in SCs such as Yap, decreased their expression following chronic EMF exposure.

In the present paper, NF2 expression is increased by chronic EMF exposure. This effect, opposite to what was previously observed in rat, is quite intriguing and might be ascribed to the different species and/or to the different exposure protocol (acute vs. chronic). The HEI-193 cell line was established from a NF2 patient with a specific mutation leading to a truncated merlin form that is distinct from that of naïve rat SCs. It is feasible that VS undergoes a kind of compensatory protective mechanism, trying to restore the oncosupressor merlin in response to the chronic EMF exposure. However, it is evident that chronic EMF exposure differently regulates the expression of some genes related to HL. In particular, genes such as *NEFL*, *TPRN*, *HMOX1*, and *OTOGL* as well as *GJB2* and *REST* were up- or downregulated, respectively, following chronic EMF. The protein taperin, a sensory epithelia protein encoded by the *TPRN* gene as well as the protein encoded by *OTOGL*, are characteristic of the nonsyndromic HL [36]. Similarly, *GJB2*, coding for the Cx32 protein expressed by SCs and present at the paranodal location [37] and *REST* are involved in nonsyndromic HL [38,39]. Interestingly, *HMOX1*, coding for the protein heme oxygenase1, possesses an emerging role in regulating oxidative stress in SCs during nerve degeneration [40]. Overall, these data provide intriguing insights into differential gene expression associated to EMF exposure in an established cell model of VS and justify further analysis to confirm changes in HL genes.

Herein, we hypothesize that EMF exposure represents a second hit, affecting SC development in pre-constituted susceptible human subjects (bearing NF2 mutations or changes in merlin expression) prone to developing VS and subsequent HL. The impact of EMF on cells and organisms has long been discussed. Data collected from case–control and case–case studies suggested the pathogenic association of EMF exposure with the increased risk to develop VS and HL [12,41,42,43]. Specific EMF effects on several cellular parameters such as cell migration, cytoskeleton reorganization, ion channel regulation, and oxidative balance have been proposed [44,45,46]. However, due to differences in the experimental protocols used for EMF exposure, these data are difficult to compare. Non-ionizing radiation by mobile phones showed a slight increase in brain tissue temperature [47,48] and has been associated with an imbalance of reactive oxygen species production [44]. This toxic mechanism might produce an increase in the blood–brain barrier permeability, leading to a deregulation of several signaling pathways [44]. However, to our knowledge, no consistent experimental data on the mechanisms linking EMF exposure to VS induction has been published. One study analyzing putative biological changes in SCs exposed to EMF suggested a weak increase in the proliferation rate, but not substantial morphological alterations [49]. Unfortunately, these authors did not consider any other parameter related to SC differentiation and/or myelinating capability. We used an established protocol [50], which showed that the application of 50 Hz, 1 mT EMF for different exposure times, induced some effects on brain cell functions. This protocol was adapted to our VS cell cultures. The EMF intensity utilized in our study was higher than those produced by common electronic devices or household electrical equipment (see for reference www.emf-portal.org, access on 16 July 2021), thus compelling cells to their maximal adaptive response. We should also consider that population is simultaneously exposed to multiple EMF sources. Nonetheless, it is noteworthy that very-low frequency EMFs have been proposed as helpful tools to promote the nerve regeneration [51,52]; however, there is inadequate data on the potential risks of low frequency EMFs for human health.

In conclusion, our findings suggest that chronic EMF exposure might be deleterious for VS at a pre-clinical stage, and could promote the transformation of VS cells toward an HL phenotype. It should be highlighted that about 50% of patients develop VS on the right side, whereas the overall population is represented by 70% right-handed. We do not exclude that other pathogenic mechanisms should be considered, or might be involved in the EMF exposure. Although further experiments are needed to explore a more direct cause–effect correlation between mobile exposure and VS pathogenesis, for precautionary purposes, subjects potentially predisposed to developing VS should pay more attention to low frequency EMF exposure.

## Figures and Tables

**Table 1:** Major signaling pathways upregulated in HEI-193 cells following chronic EMF exposure.

Pathway		Direction	NES	*p* adj
	Cotranslational protein targeting to membrane	Up	7.859	5.30 × 10^−10^
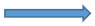	Protein targeting to ER	Up	7.807	5.30 × 10^−10^
	SRP dependent cotranslational protein targeting to membrane	Up	7.797	5.30 × 10^−10^
	Establishment of protein localization to endoplasmic reticulum	Up	7.585	5.30 × 10^−10^
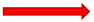	Mitochondrial translational elongation	Up	6.081	6.10 × 10^−6^
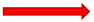	Mitochondrial translational termination	Up	6.022	6.70 × 10^−6^
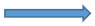	Translational termination	Up	5.668	1.50 × 10^−5^
	Mitochondrial respiratory chain complex assembly	Up	4.546	9.00 × 10^−4^
	Mitochondrial ATP synthesis coupled electron transport	Up	4.404	1.20 × 10^−3^
	Cytoplasmic translation	Up	4.360	1.50 × 10^−3^
	ATP synthesis coupled electron transport	Up	4.234	1.90 × 10^−3^
	NADH dehydrogenase complex assembly	Up	3.955	6.60 × 10^−3^
	Mitochondrial respiratory chain complex I assembly	Up	3.955	6.60 × 10^−3^
	Ribosomal large subunit biogenesis	Up	3.920	6.60 × 10^−3^
	Respiratory electron transport chain	Up	3.858	6.60 × 10^−3^
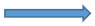	Ribosome assembly	Up	3.414	3.10 × 10^−2^

## Data Availability

RNA-Seq files (Fastq data and quantitation matrix) were deposited in the GEO (gene expression omnibus) database. The access number is GSE174389 and the security token is ixovwimkrlebdkf.

## Supplementary Materials

The following are available online at https://www.mdpi.com/article/10.3390/cells10071840/s1, Figure S1: Heat map of the up- and downregulated genes in HEI193 cells from CTRL versus EMF exposed cells. DEGs were selected setting a 2-fold change, Figure S2: Heat map of the up- and downregulated genes in HEI193 cells from CTRL versus EMF exposed cells. DEGs were selected setting a 1.5-fold change.
