# Supplementary figures and images for: Transcriptomic Profile Reveals Deregulation of Hearing-Loss Related Genes in Vestibular Schwannoma Cells Following Electromagnetic Field Exposure
Source: Cells. 2021 Jul 20;10(7):1840. doi: 10.3390/cells10071840 (PMC8307028; doi:10.3390/cells10071840)

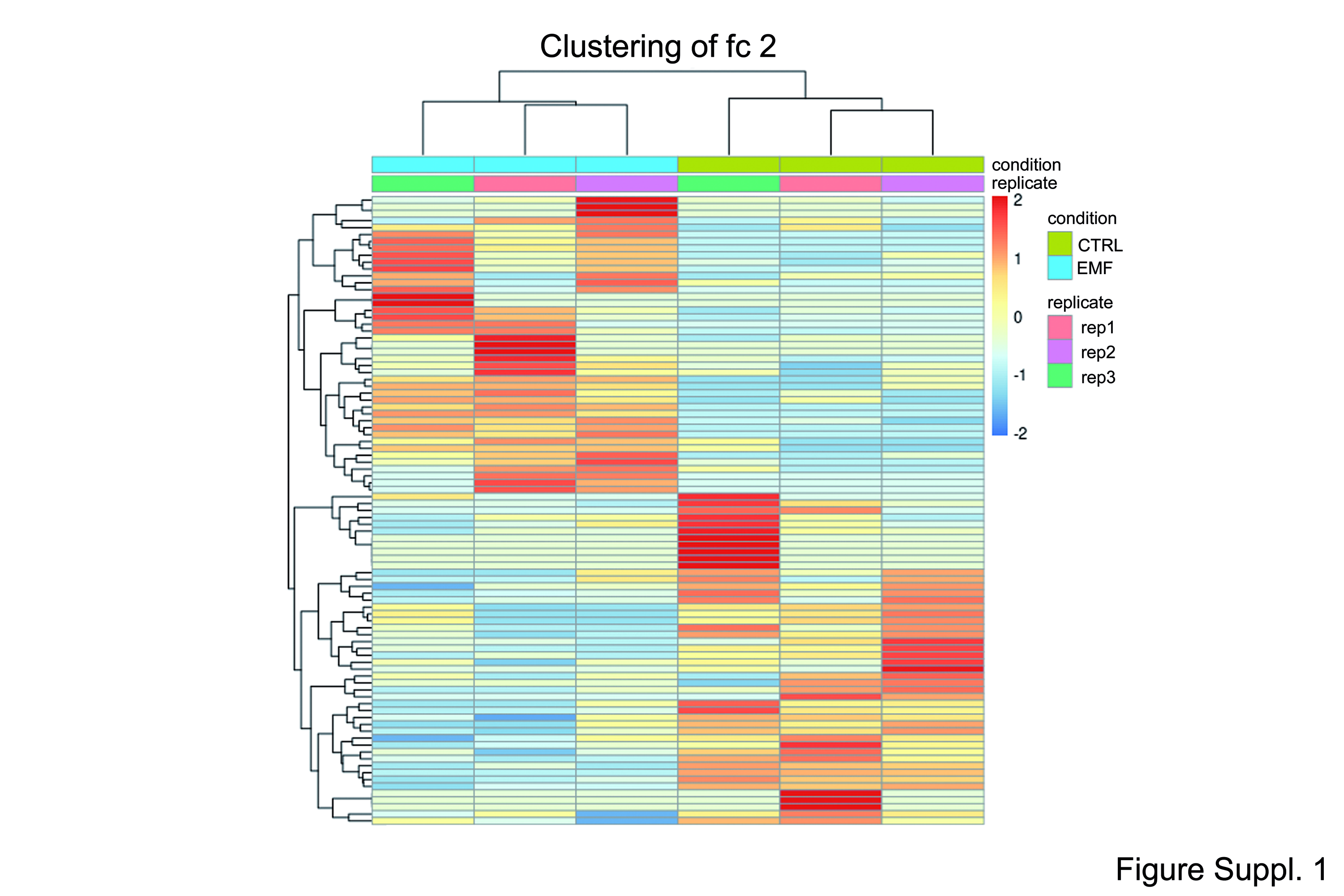

Supplement: Supplementary file 1 [file cells-10-01840-s001.zip › cells-1275568-supplementary/FigSuppl1.tif]

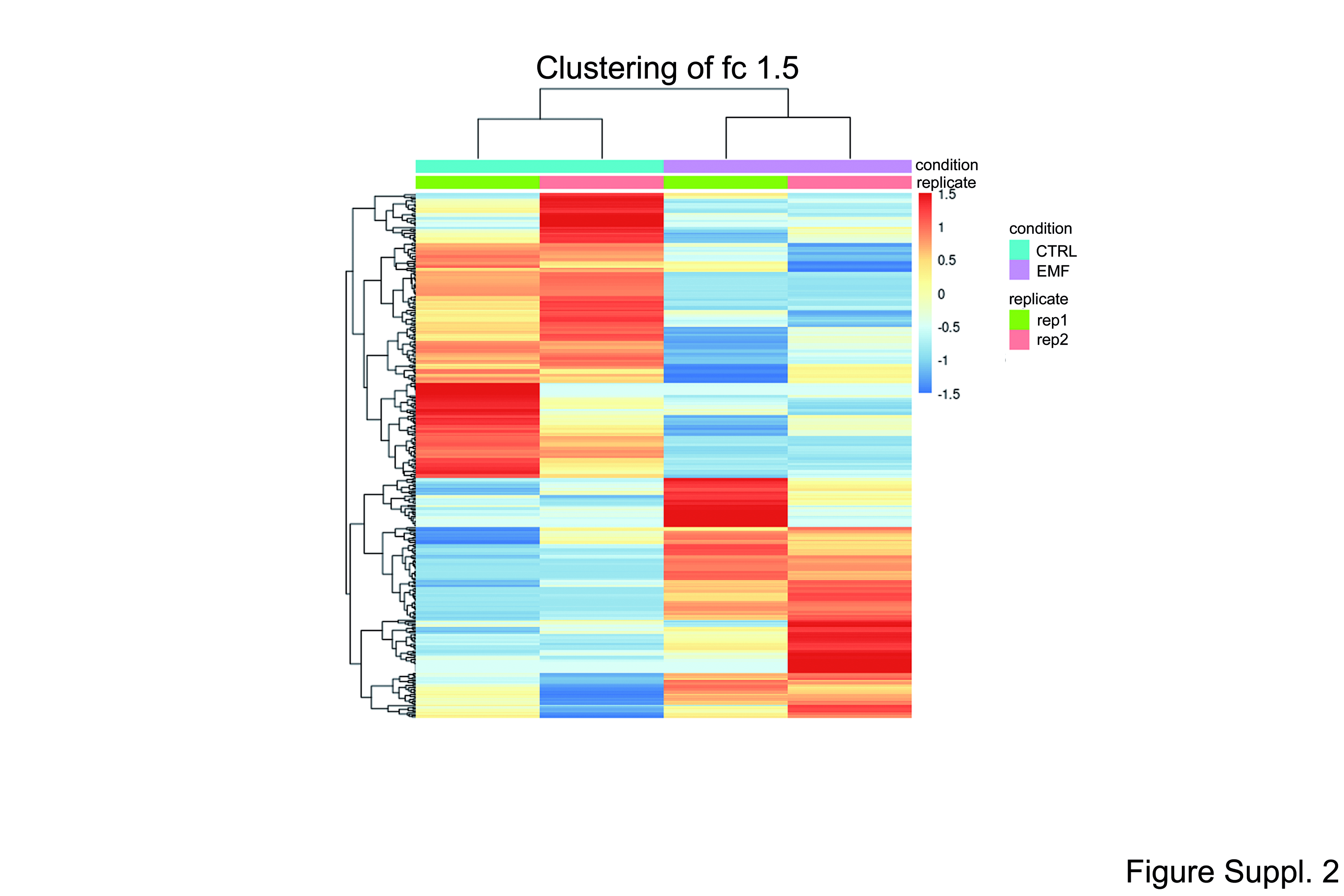

Supplement: Supplementary file 1 [file cells-10-01840-s001.zip › cells-1275568-supplementary/FigSuppl2.tif]
